# A Comprehensive Echocardiographic Assessment of Neonatal Right Ventricular Function in Neonatal Intensive Care Unit Babies

**DOI:** 10.7759/cureus.37166

**Published:** 2023-04-05

**Authors:** Nikita Khot, Santosh Joshi, Sudhir Malwade, Sanjay Chavan, Shailaja V Mane, Sharad Agarkhedkar, Amodini Arora

**Affiliations:** 1 Pediatrics, Dr. D. Y. Patil Medical College, Hospital & Research Centre, Pune, IND; 2 Pediatric Cardiology, Dr. D. Y. Patil Medical College, Hospital & Research Centre, Pune, IND; 3 Pediatric Medicine, Dr. D. Y. Patil Medical College, Hospital & Research Centre, Pune, IND

**Keywords:** myocardial performance index, tricuspid annular plane systolic excursion, 2d echo, tapse, tricuspid inflow velocity, mpi, rv function

## Abstract

Background

The right ventricle (RV) in the fetus is the predominant chamber, accounting for about 60% of the total cardiac output. The majority of the RV outflow volume is diverted from the pulmonary artery via the ductus arteriosus to the descending aorta. After birth, the RV undergoes extensive structural and functional modifications. The RV undergoes an improper transition from fetal to neonatal circulation in sick neonatal intensive care unit (NICU) babies. Functional echocardiography is now commonly being used in most NICUs as it is a noninvasive and bedside investigation that gives an immediate evaluation of hemodynamics and can be taken into consideration as an extension of clinical assessment to study a critically unwell neonate. Therefore, a study of RV functions in NICU neonates will help in better understanding the neonatal cardiopulmonary response to different diseases. Thus, this study aimed to assess RV functions in neonates getting admitted to the NICU of a tertiary care institute.

Methodology

This observational, cross-sectional study was approved by the Research & Recognition Committee of Dr. D. Y. Patil Vidyapeeth, Pune. In total, 35 cases of term neonates admitted to the NICU at Dr. D. Y. Patil Medical College, Hospital & Research Centre, Pune who fulfilled the inclusion criteria were enrolled in this study after obtaining consent from their parents. Two-dimensional echocardiography was performed by a trained pediatric cardiologist, and the findings were substantiated by a neonatologist trained in echocardiography.

Results

Our study found a strong association between tricuspid inflow velocity and neonates with sepsis. Similarly, a significant association was observed between abnormal tricuspid Inflow velocity (E/A and E/E’) and neonates requiring inotropic support.

Conclusions

Data on the normal values of different echocardiographic parameters of the systolic and diastolic function of the RV during the neonatal phase of life are currently limited. Our data offer preliminary insights into this topic. Early echocardiography and intervention are advisable, especially in neonates with sepsis and requiring inotropic support.

## Introduction

The cardiovascular system of neonates is not a miniature version of the mature human body. The fetal heart is functionally adapted to a low-pressure environment with high pulmonary blood flow and low systemic vascular resistance [1]. For a successful transition from intrauterine to extrauterine life, birth requires a variety of circulatory changes. In addition to the radical adaptation to an extrauterine circulation after delivery, the newborn heart must adapt promptly to a dual circulation with much greater systemic vascular resistance and increased metabolic needs [1].

The early indicators of altered function in the fetal heart are now believed to be a reflection of right heart hemodynamics. Observations indicate that the right ventricle (RV) of the embryonic heart contributes significantly to the work output of the fetal myocardium due to the tremendous volume and pressure work necessitated by it.

Echocardiography is a vital investigative tool in the neonatal intensive care unit (NICU), which immensely facilitates the evaluation of the neonate. Recently published studies have provided evidence that demonstrates the significance of using echocardiography in the treatment of critically ill patients. According to studies, clinical therapy is altered in 30-60% of patients after point-of-care echocardiography, and this technique is currently regarded as an essential tool in the management of sick neonates [2,3].

Tricuspid annular plane systolic excursion (TAPSE) or S’ wave on tissue Doppler imaging are good reflectors of RV systolic function and can be reliably performed using bedside functional echocardiography [4,5]. It measures the excursion of the tricuspid annulus during the cardiac cycle (between early diastole and end systole). A relatively new measurement of ventricular function, the myocardial performance index (MPI), has been shown to correlate with other quantifiable, noninvasive, and invasive measures of ventricular function [6-10]. The MPI is a ratio and is derived by dividing the total time spent in isovolumic activity (isovolumic contraction time and isovolumic relaxation time) by the time spent in ventricular ejection.

The left ventricle has been more widely studied compared to the RV. In our view, the RV is equally important because, after birth, the RV undergoes an improper transition from fetal to neonatal circulation in sick NICU babies. Therefore, a study of RV functions in NICU neonates will help in better understanding the neonatal cardiopulmonary response to different diseases. Thus, this study aimed to assess RV functions in neonates admitted to the NICU of a tertiary care institute.

## Materials and methods

In this observational, cross-sectional study, 35 neonates who were admitted to the Level III NICU of Dr. D Y Patil Medical College, Hospital & Research Centre, Pimpri during two years were evaluated with bedside echocardiography to assess RV function. The study was conducted after obtaining approval from the Research & Recognition Committee (approval ID: IESC/PGS/2020/40) and informed consent from parents. Two-dimensional echocardiography was performed by a trained pediatric cardiologist, and the findings were substantiated by a neonatologist trained in echocardiography. Two-dimensional echocardiography was performed using Fujifilm Sonosite Edge II (serial number: Q53FGW), which was calibrated regularly.

After birth, the RV undergoes extensive structural and functional modifications. There is a progressive decrease in RV wall thickness and growth in left ventricular mass after birth due to the marked fall in pulmonary vascular resistance (PVR) and increased systemic vascular resistance (SVR). Unlike modifications in the RV during the normal transition, RV hypertrophy persists in case of sustained elevations of PVR, persistent pulmonary hypertension of the newborn, congenital coronary heart disease, and other cardiopulmonary disorders. RV adaptation and/or maladaptation may play a role in the clinical condition of the baby and needs to be studied in detail. Hence, this study would help us understand the underlying RV dysfunction of the neonate and its impact on the clinical outcome.

Term neonates admitted to NICU at Dr. D Y Patil Medical College, Hospital & Research Centre, Pimpri needing oxygen therapy, inotropic support, prolonged antibiotic therapy, and/or exchange transfusion were included in the study. Preterm babies, babies with congenital heart disease, and babies of HIV-positive mothers were excluded from the study. RV function was assessed by measuring TAPSE, tricuspid inflow velocities (E/A and E/E’), and MPI. Term neonates admitted to the NICU who fulfilled the inclusion criteria were included in this study. The detailed study procedure was explained to the parents of the neonates eligible for the study in a language they understood and written informed consent was taken before the commencement of the study. Data was collected in a preformed data collection form and entered in Microsoft Excel. The study statistics are presented in the form of numbers and percentages for qualitative data and quantitative data. Mean and standard deviation were used. Appropriate statistical tests such as the chi-square test and p-value were applied. SPSS software version 26 (IBM Corp., Armonk, NY, USA) was used for data analysis.

## Results

Of the 35 cases in this study, RV function was assessed using parameters such as tricuspid inflow velocity, RV MPI, and TAPSE and was categorized according to the etiology of admission. Table 1 shows the distribution of study participants according to etiology. However, an overlap of etiology was seen in some patients.

**Table 1:** Distribution of study participants according to etiology.

Etiology	N (35)	%
Meconium aspiration syndrome	20	57.1
Perinatal hypoxia/depression with encephalopathy	11	31.4
Perinatal hypoxia/depression without encephalopathy	03	8.6
Sepsis and congenital defects	03	8.6
Sepsis and neonatal seizures	08	22.9
Pathological jaundice	06	17.1

The most common etiology observed for NICU admission was meconium aspiration syndrome (57.1%), followed by perinatal hypoxia (40%), sepsis (31.5%), and pathological jaundice (17.1%).

The RV function was assessed using various modalities such as TAPSE, tricuspid inflow velocities, and MPI, as shown in Table 2.

**Table 2:** Assessment of RV function using various modalities. TAPSE: tricuspid annular plane systolic excursion; RV: right ventricle

Parameters	Mean ± SD (observed)	Mean ± SD (normal)
Tricuspid E/A ratio	01.14 ± 0.66	0.88 ± 0.17
Tricuspid E/E’ ratio	06.40 ± 2.41	6.2 ± 1.5
TAPSE	10.87 ± 2.69	10.6 ± 4.0
RV myocardial performance index	0.3404 ± 0.185	0.5 ± 0.1

In our study, most cases had observed values near the reference range except tricuspid inflow velocity (E/A), which were lower than the reference range, and RV MPI was observed to be higher than the reference range.

Table 3 shows the RV function (assessed using tricuspid inflow velocity - E/A and E/E’) observed in our study participants along with its comparison with the reference range.

**Table 3:** Distribution of RV function (E/A ratio and E/E’ ratio) of each participant and its comparison with the reference range. RV: right ventricle; MAS: meconium aspiration syndrome; AKI: acute kidney injury; MSSA: methicillin-sensitive *Staphylococcus aureus*; CRP: C-reactive protein

Etiology	E/A		E/E’	
	Observed	Interpretation	Observed	Interpretation
Congenital diaphragmatic hernia with sepsis	0.86	Normal	4.35	Abnormal
MAS with neonatal seizures and sepsis	1.82	Abnormal	9.2	Abnormal
MAS with respiratory distress	0.81	Normal	6.2	Normal
Neonate of a diabetic mother with pathological jaundice	0.82	Normal	6.9	Normal
MAS with respiratory distress	0.88	Normal	7.02	Normal
MAS with respiratory distress with sepsis	0.84	Normal	4.51	Abnormal
MAS with respiratory distress	0.85	Normal	6.1	Normal
MAS with perinatal hypoxia with refractory hypoglycemia	0.57	Abnormal	3.8	Abnormal
Neonate of a diabetic mother with MAS with pathological jaundice	0.93	Normal	10.7	Abnormal
Perinatal hypoxia with neonatal encephalopathy	0.87	Normal	8.48	Abnormal
Perinatal hypoxia with respiratory distress	0.96	Normal	6.8	Normal
Pathological jaundice	0.75	Normal	4.9	Normal
MAS with pathological jaundice	0.84	Normal	4.86	Normal
Perinatal hypoxia with pathological jaundice	0.78	Normal	5.3	Normal
Perinatal hypoxia with pathological jaundice	0.76	Normal	6.3	Normal
MAS with respiratory distress	0.68	Abnormal	4.9	Normal
MAS with respiratory distress	0.62	Abnormal	3.4	Abnormal
Neonatal seizures with right-sided pneumothorax with CRP-positive sepsis	1.11	Abnormal	6.4	Normal
Perinatal hypoxia with neonatal encephalopathy stage 2	2.97	Abnormal	4.8	Normal
MAS with perinatal hypoxia	2.8	Abnormal	7.2	Normal
MAS with perinatal hypoxia with neonatal encephalopathy	0.79	Normal	4.07	Abnormal
Rotavirus encephalitis with MSSA sepsis	0.79	Normal	3.11	Abnormal
Perinatal hypoxia with no signs of encephalopathy	1.15	Abnormal	12.16	Abnormal
MAS with respiratory distress	0.79	Normal	4.8	Normal
Perinatal hypoxia with hypotonia with MAS with the neonate of a diabetic mother	1.64	Abnormal	5.77	Normal
MAS with respiratory distress	0.79	Normal	7.35	Normal
MAS with perinatal hypoxia with stage 2 encephalopathy with neonatal seizures	2.9	Abnormal	5.04	Normal
Perinatal hypoxia with neonatal encephalopathy	2.1	Abnormal	3.9	Abnormal
MAS with respiratory distress	0.81	Normal	6.8	Normal
*Enterococcus faecalis* sepsis with splenic and renal cyst with AKI on peritoneal dialysis	1.66	Abnormal	12	Abnormal
Perinatal hypoxia with neonatal encephalopathy	1.59	Abnormal	10.8	Abnormal
Neonatal seizures with *Acinetobacter *sepsis	1.27	Abnormal	9.08	Abnormal
MAS with respiratory distress	0.61	Normal	8.2	Abnormal
MAS with respiratory distress with pathological jaundice	0.7	Normal	4.3	Abnormal
MAS with perinatal hypoxia with neonatal encephalopathy	0.68	Abnormal	4.4	Abnormal

Table 4 shows the RV function (assessed using TAPSE and MPI) observed in our study participants along with its comparison with the reference range.

**Table 4:** Distribution of RV function (TAPSE and MPI) of each participant and its comparison with the reference ranges. NICU: neonatal intensive care unit; MAS: meconium aspiration syndrome; TAPSE: tricuspid annular plane systolic excursion; MPI: myocardial performance index; AKI: acute kidney injury; MSSA: methicillin-sensitive *Staphylococcus aureus*; CRP: C-reactive protein

Reason for NICU admission	TAPSE		MPI	
	Observed	Interpretation	Observed	Interpretation
Congenital diaphragmatic hernia with sepsis	6.4	Abnormal	0.56	Normal
MAS with neonatal seizures and sepsis	7.8	Normal	0.19	Abnormal
MAS with respiratory distress	9.8	Normal	0.31	Abnormal
Neonate of a diabetic mother with pathological jaundice	11.3	Normal	0.08	Abnormal
MAS with respiratory distress	10.9	Normal	0.31	Abnormal
MAS with respiratory distress with sepsis	9.5	Normal	0.3	Abnormal
MAS with respiratory distress	7.6	Normal	0.5	Normal
MAS with perinatal hypoxia with refractory hypoglycemia	8.8	Normal	0.49	Normal
Neonate of a diabetic mother with MAS with pathological jaundice	11.7	Normal	0.3	Abnormal
Perinatal hypoxia with neonatal encephalopathy	11.9	Normal	0.44	Normal
Perinatal hypoxia with respiratory distress	8.7	Normal	0.43	Normal
Pathological jaundice	10.8	Normal	0.56	Normal
MAS with pathological jaundice	9.7	Normal	0.3	Abnormal
Perinatal hypoxia with pathological jaundice	11.3	Normal	0.14	Abnormal
Perinatal hypoxia with pathological jaundice	10.8	Normal	0.33	Abnormal
MAS with respiratory distress	10.4	Normal	0.36	Abnormal
MAS with respiratory distress	8.9	Normal	0.83	Abnormal
Neonatal seizures with right-sided pneumothorax with CRP-positive sepsis	14.9	Abnormal	0.29	Abnormal
Perinatal hypoxia with neonatal encephalopathy stage 2	15.2	Abnormal	0.08	Abnormal
MAS with perinatal hypoxia	10	Normal	0.1	Abnormal
MAS with perinatal hypoxia with neonatal encephalopathy	8.3	Normal	0.19	Abnormal
Rotavirus encephalitis with MSSA sepsis	10.6	Normal	0.12	Abnormal
Perinatal hypoxia with no signs of encephalopathy	12	Normal	0.35	Abnormal
MAS with respiratory distress	10.2	Normal	0.31	Abnormal
Perinatal hypoxia with hypotonia with MAS with neonate of a diabetic mother	11.8	Normal	0.48	Normal
MAS with respiratory distress	11.2	Normal	0.41	Normal
MAS with perinatal hypoxia with stage 2 encephalopathy with neonatal seizures	15.3	Abnormal	0.15	Abnormal
Perinatal hypoxia with neonatal encephalopathy	16.2	Abnormal	0.27	Abnormal
MAS with respiratory distress	11.1	Normal	0.7	Abnormal
*Enterococcus faecalis* sepsis with splenic and renal cyst with AKI on peritoneal dialysis	10	Normal	0.36	Abnormal
Perinatal hypoxia with neonatal encephalopathy	5.9	Normal	0.414	Normal
Neonatal seizures with *Acinetobacter *sepsis	15.9	Abnormal	0.261	Abnormal
MAS with respiratory distress	10.4	Normal	0.146	Abnormal
MAS with respiratory distress with pathological jaundice	16.9	Abnormal	0.671	Abnormal
MAS with perinatal hypoxia with neonatal encephalopathy	8.3	Normal	0.156	Abnormal

Table 5 depicts the RV function observed in different etiologies. There was no statistical association between tricuspid inflow velocity (E/A Ratio) and neonates with pathological jaundice. A strong association was noted between abnormal tricuspid inflow velocity (E/A Ratio) and neonates with sepsis (four out of six abnormal values were increased). It was observed that other etiologies and right ventricular function did not have a statistical correlation.

**Table 5:** Association of RV function with etiology. Using the chi-square test; *: p <0.05 is significant. RV: right ventricle; TAPSE: tricuspid annular plane systolic excursion; E/A and E/E’: tricuspid inflow velocity; MPI: myocardial performance index

Etiology	E/A (n = 35)		P-values
	Normal (%) (N = 20)	Abnormal (%) (N = 15)	
Meconium aspiration syndrome	12 (60.0)	08 (53.3)	0.693
Perinatal hypoxia with encephalopathy	04 (20.0)	07 (46.7)	0.093
Perinatal hypoxia without encephalopathy	01 (05.0)	02 (13.3)	0.383
Pathological jaundice	06 (30.0)	-	0.020*
Sepsis and congenital defects	02 (10.0)	01 (06.7)	0.727
Sepsis and neonatal seizures	02 (10.0)	06 (40.0)	0.036*
Etiology	E/E’ (n = 35)		P-values
	Normal (%) (N = 18)	Abnormal (%) (N = 17)	
Meconium aspiration syndrome	11 (61.1)	09 (52.9)	0.625
Perinatal hypoxia with encephalopathy	05 (27.8)	06 (35.3)	0.632
Perinatal hypoxia without encephalopathy	02 (11.1)	01 (05.9)	0.581
Pathological jaundice	04 (22.2)	02 (11.8)	0.412
Sepsis and congenital defects	-	03 (17.6)	0.062
Sepsis and neonatal seizures	03 (16.7)	05 (29.4)	0.369
Etiology	TAPSE (n = 35)		P-values
	Normal (%) (N = 28)	Abnormal (%) (N = 07)	
Meconium aspiration syndrome	18 (64.3)	02 (28.6)	0.088
Perinatal hypoxia with encephalopathy	08 (28.6)	03 (42.9)	0.466
Perinatal hypoxia without encephalopathy	03 (10.7)	-	0.365
Pathological jaundice	05 (17.9)	01 (14.3)	0.823
Sepsis and congenital defects	02 (07.1)	01 (14.3)	0.546
Sepsis and neonatal seizures	05 (17.9)	03 (42.9)	0.159
Etiology	MPI (n = 35)		P-values
	Normal (%) (N = 09)	Abnormal (%) (N = 26)	
Meconium aspiration syndrome	04 (44.4)	16 (61.5)	0.372
Perinatal hypoxia with encephalopathy	04 (44.4)	07 (26.9)	0.329
Perinatal hypoxia without encephalopathy	01 (11.1)	02 (07.7)	0.752
Pathological jaundice	01 (11.1)	05 (19.2)	0.577
Sepsis and congenital defects	01 (11.1)	02 (07.7)	0.752
Sepsis and neonatal seizures	-	08 (30.8)	0.058

Table 6 depicts the association of the RV function with inotropic support. A statistically significant association was observed between abnormal tricuspid inflow velocity (E/A and E/E’) and neonates requiring inotropic support with a significant p-value of 0.005 and 0.053, respectively. It was observed that among those having abnormal E/A ratio, most were increased, whereas the majority of those having abnormal E/E’ had decreased values. Of those on inotropic support, there was a statistically significant association between tricuspid inflow velocity (E/A) and neonates on monotherapy inotropic support with a significant p-value of 0.040. Similarly, there was no association between TAPSE and neonates on polytherapy inotropic support with a significant p-value of 0.011. Early inotropic support may be beneficial for the overall outcome/condition of the baby.

**Table 6:** Association of RV function with inotropic support. Using the chi-square test; *: p <0.05 is significant. RV: right ventricle; E/A and E/E’: tricuspid inflow velocity; MPI: myocardial performance index; TAPSE: tricuspid annular plane systolic excursion

RV functions	Inotropic support (n = 35)		P-values
	Not required (%) (N = 14)	Required (%) (N = 21)	
E/A			0.005*
Normal	12 (85.7)	08 (38.1)	
Abnormal	02 (14.3)	13 (61.9)	
E/E’			0.053*
Normal	10 (71.4)	08 (38.1)	
Abnormal	04 (28.6)	13 (61.9)	
TAPSE			0.121
Normal	13 (92.9)	15 (71.4)	
Abnormal	01 (07.1)	06 (28.6	
MPI			0.636
Normal	03 (21.4)	06 (28.6)	
Abnormal	11 (78.6)	15 (71.4)	
RV functions	Monotherapy support (n = 35)		P-values
	Not required (%) (N = 25)	Required (%) (N = 10)	
E/A			0.040*
Normal	17 (68.0)	03 (30.0)	
Abnormal	08 (32.0)	07 (70.0)	
E/E’			0.392
Normal	14 (56.0)	04 (40.0)	
Abnormal	11 (44.0)	06 (60.0)	
TAPSE			0.350
Normal	19 (76.0)	09 (90.0)	
Abnormal	06 (24.0)	01 (10.0)	
MPI			0.625
Normal	07 (28.0)	02 (20.0)	
Abnormal	18 (72.0)	08 (80.0)	
RV functions	Polytherapy support (n = 35)		P-values
	Not required (%) (N = 24)	Required (%) (N = 11)	
E/A			0.344
Normal	15 (62.5)	05 (45.5)	
Abnormal	09 (37.5)	06 (54.5)	
E/E’			0.227
Normal	14 (58.3)	04 (36.4)	
Abnormal	10 (41.7)	07 (63.6)	
TAPSE			0.011*
Normal	22 (91.7)	06 (54.5)	
Abnormal	02 (08.3)	05 (45.5)	
MPI			0.329
Normal	05 (20.8)	04 (36.4)	
Abnormal	19 (79.2)	07 (63.6)	

## Discussion

The role of echocardiography in the NICU has changed over the past few years. More recently, neonatologists have become interested in the echocardiographic assessment of hemodynamic instability in infants. The terms functional echocardiography and point-of-care echocardiography have been introduced to describe the use of echocardiography as an adjunct in the clinical assessment of the hemodynamic status in neonates. The increasing availability of echocardiography, with the miniaturization of the technology, has resulted in the more widespread use of echocardiography in NICUs globally. In addition, newborns in the NICU are unique as they are in the process of transition from fetal to postnatal circulation.

**Figure 1:**
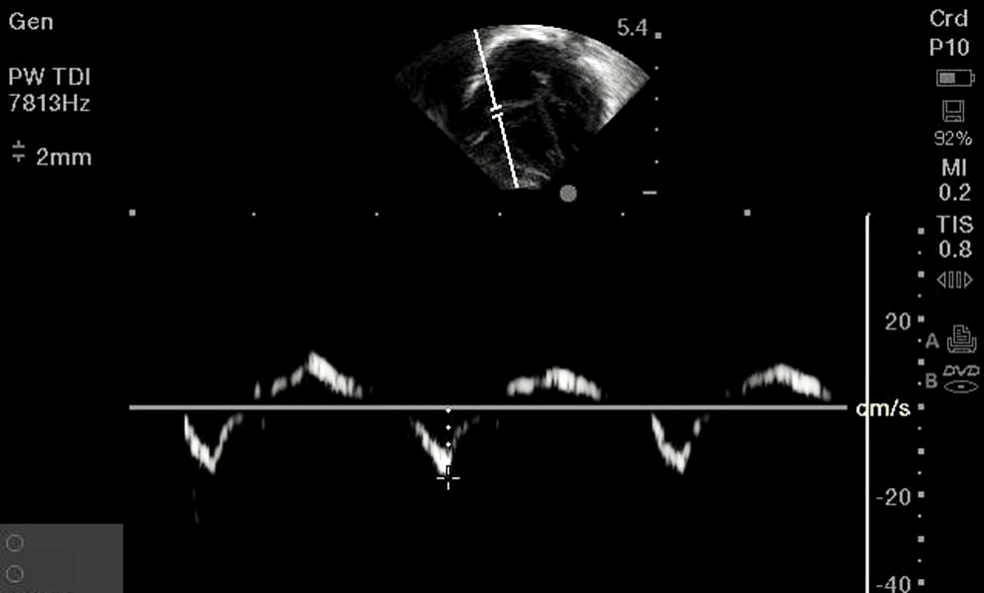
Image shows the tricuspid inflow velocity (E’) values.

In our study, the most common etiology observed for NICU admission was meconium aspiration syndrome (57.1%), followed by perinatal hypoxia (40.3%), sepsis (31.5%), and pathological jaundice (17.1%). Most study participants had observed values near the reference range except tricuspid inflow velocity (E/A), which was found to be lower than the reference range, and RV MPI, which was observed to be higher than the reference range. The reference ranges were reported in the study by Pedraza-Melchor et al. who assessed the RV anatomical and functional parameters in healthy term neonates, by Mah et al. who assessed the RV function of patients with pediatric heart disease, and by Smith et al. who assessed the RV function of healthy term neonates [11-13]. Figure 1 shows the assessment of the RV function by tricuspid inflow velocity (E’ value).

**Figure 2:**
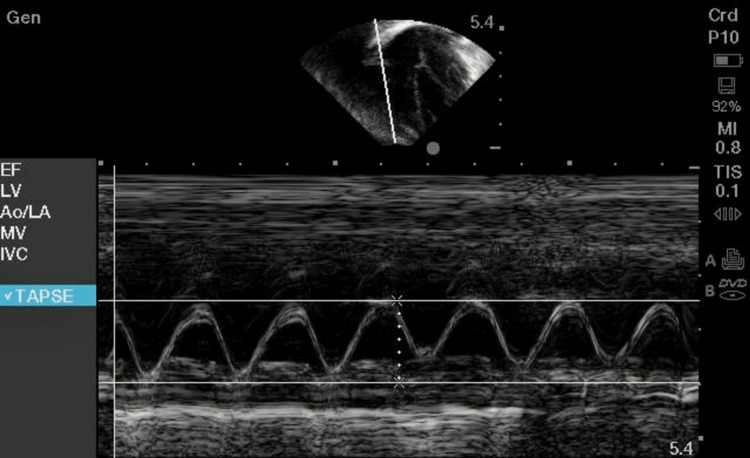
M mode tracing showing tricuspid annulus plane systolic excursion.

Figure 2 shows the assessment of the RV function by TAPSE using M mode in two-dimensional echocardiography.

**Figure 3:**
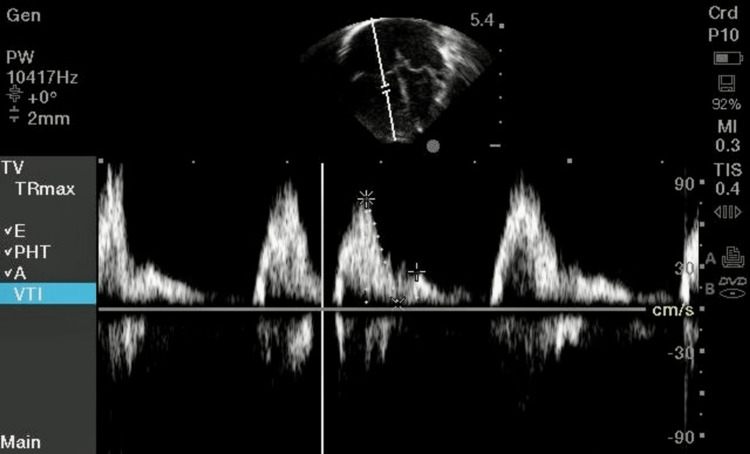
Imaging shows the tricuspid inflow velocity (E/A) values of the tricuspid valve.

In our study, a strong association was noted between tricuspid inflow velocity and neonates with sepsis (six abnormal values were observed, of which four were increased). Vallabhajosyula et al. studied RV dysfunction in septic patients. It was found that isolated RV dysfunction is seen commonly in sepsis and septic shock and is associated with worse long-term survival. RV dysfunction in sepsis is multifactorial and can be caused by direct myocardial depression, hemodynamic derangements or an increase in RV afterload due to hypoxemia, hypercapnia, and mechanical ventilation for acute respiratory failure [11]. Sobeih et al. evaluated the echocardiographic parameters in 20 neonates with perinatal asphyxia (cases) and 20 healthy full-term non-asphyxiated neonates (controls). Among pulsed-wave Doppler parameters, cases had statistically significant lower values than controls for mitral E velocity and mitral E/A ratio. Similarly, among tissue Doppler parameters, cases had statistically significant lower values than controls for septal E’/A’ ratio, left ventricular E’ velocity, and E’/A’ ratio [14]. Peček et al. quantitatively characterized changes in RV function using echocardiography in 35 healthy term newborns between the third and the seventh day of life. They concluded that increased RV systolic and diastolic myocardial velocities, cardiac output and longitudinal deformation, and decreased RV MPI’ between the third and the seventh day of life point to a reduction of RV afterload and adaptive myocardial maturation in term newborns during this period. Hence, early echocardiography and intervention may help in the overall clinical outcomes of the patient [15]. Figure 3 shows the assessment of the RV function by tricuspid inflow velocity (E/A value).

Our study found no statistical association between tricuspid inflow velocity (E/A ratio) and neonates with pathological jaundice. There was no statistically significant correlation between RV function and clinical outcomes. However, it was observed that of the neonates who died, 66.7% had abnormal E/A and 100% had abnormal E/E’ values. Hence, emphasizing the importance of early diagnosis of abnormal RV function and intervention would lead to better clinical outcomes.

The limitations of the study were the limited literature available on RV function in neonates. Moreover, the data analysis was based on a relatively small number of patients in a single center, which might have limited the power to detect subtle cardiac dysfunction.

## Conclusions

Currently, there is insufficient data on the normal values of various echocardiographic parameters of the systolic and diastolic function of the RV in the neonatal phase of life. During this period, the RV relaxation pattern is impaired. This may be the result of only a partial regression of RV predominance in fetal circulation. Our data offer preliminary insights into this topic. It is advisable to perform echocardiography for all neonates admitted to the NICU within 48 hours of birth, especially those having sepsis.

## References

[1] Marijianowski MM, van der Loos CM, Mohrschladt MF, Becker AE (1994). The neonatal heart has a relatively high content of total collagen and type I collagen, a condition that may explain the less compliant state. J Am Coll Cardiol.

[2] Ranjit S, Aram G, Kissoon N (2014). Multimodal monitoring for hemodynamic categorization and management of pediatric septic shock: a pilot observational study*. Pediatr Crit Care Med.

[3] Manasia AR, Nagaraj HM, Kodali RB (2005). Feasibility and potential clinical utility of goal-directed transthoracic echocardiography performed by noncardiologist intensivists using a small hand-carried device (SonoHeart) in critically ill patients. J Cardiothorac Vasc Anesth.

[4] Jain A, Mohamed A, El-Khuffash A (2014). A comprehensive echocardiographic protocol for assessing neonatal right ventricular dimensions and function in the transitional period: normative data and z scores. J Am Soc Echocardiogr.

[5] Breatnach CR, Levy PT, James AT, Franklin O, El-Khuffash A (2016). Novel echocardiography methods in the functional assessment of the newborn heart. Neonatology.

[6] Tei C (1995). New non-invasive index for combined systolic and diastolic ventricular function. J Cardiol.

[7] Tei C, Ling LH, Hodge DO (1995). New index of combined systolic and diastolic myocardial performance: a simple and reproducible measure of cardiac function--a study in normals and dilated cardiomyopathy. J Cardiol.

[8] Tei C, Dujardin KS, Hodge DO, Kyle RA, Tajik AJ, Seward JB (1996). Doppler index combining systolic and diastolic myocardial performance: clinical value in cardiac amyloidosis. J Am Coll Cardiol.

[9] Tei C, Dujardin KS, Hodge DO, Bailey KR, McGoon MD, Tajik AJ, Seward SB (1996). Doppler echocardiographic index for assessment of global right ventricular function. J Am Soc Echocardiogr.

[10] Tei C, Nishimura RA, Seward JB, Tajik AJ (1997). Noninvasive Doppler-derived myocardial performance index: correlation with simultaneous measurements of cardiac catheterization measurements. J Am Soc Echocardiogr.

[11] Pedraza-Melchor RL, Hernández-Benítez R, Iglesias-Leboreiro J, Vidaña-Pérez D, Bernardez-Zapata I, Singh Y (2021). Right ventricular anatomical and functional parameters in healthy Mexican term newborns. Arch Cardiol Mex.

[12] Mah K, Mertens L (2022). Echocardiographic assessment of right ventricular function in pediatric heart disease: a practical clinical approach. CJC Pediatr Congenital Heart Dis.

[13] Smith A, Purna JR, Castaldo MP (2019). Accuracy and reliability of qualitative echocardiography assessment of right ventricular size and function in neonates. Echocardiography.

[14] Sobeih AA, El-Baz MS, El-Shemy DM, Abu El-Hamed WA (2021). Tissue Doppler imaging versus conventional echocardiography in assessment of cardiac diastolic function in full term neonates with perinatal asphyxia. J Matern Fetal Neonatal Med.

[15] Peček J, Koželj M, Lenasi H, Fister P (2022). Right ventricular function in neonates during early postnatal period: a prospective observational study. Pediatr Cardiol.

